# Echinochrome A Improves Exercise Capacity during Short-Term Endurance Training in Rats

**DOI:** 10.3390/md13095722

**Published:** 2015-09-08

**Authors:** Dae Yun Seo, Robin A. McGregor, Su Jin Noh, Seung Jun Choi, Natalia P. Mishchenko, Sergey A. Fedoreyev, Valentin A. Stonik, Jin Han

**Affiliations:** 1 National Research Laboratory for Mitochondrial Signaling, Department of Physiology, College of Medicine, Cardiovascular and Metabolic Disease Center, Inje University, Bokji-ro 75, Busanjin, Busan 633-165, Korea; E-Mails: sdy925@gmail.com (D.Y.S.); robinmcgregor@gmail.com (R.A.M.); msns6336@hanmail.net (S.J.N.); 2 Division of Sports and Health Science, Kyungsung University, 309 Suyoung-ro, Nam-gu, Busan 608-736, Korea; E-Mail: choisj@ks.ac.kr; 3 George B. Elyakov Pacific Institute of Bioorganic Chemistry, Far-Eastern Branch of the Russian, Academy of Science, Prospect 100 let Vladivostoku, 159, Vladivostok 690022, Russia; E-Mails: mischenkonp@mail.ru (N.P.M.); fedoreev-s@mail.ru (S.A.F.); stonik@piboc.dvo.ru (V.A.S.)

**Keywords:** aerobic exercise, Echinochrome A, skeletal muscle, mitochondrial function

## Abstract

Echinochrome A (Echi A) improves mitochondrial function in the heart; however, its effects on skeletal muscle are still unclear. We hypothesized that Echi A administration during short-term exercise may improve exercise capacity. Twenty-four male Sprague-Dawley rats were randomly divided into the following groups: control group (CG), Echi A-treated group (EG), aerobic exercise group (AG), and aerobic exercise treated with Echi A group (AEG) (*n* = 6 per group). Echi A was administered intra-peritoneally (0.1 mg/kg of Echi A in 300 µL phosphate-buffered saline) daily 30 min before each exercise training. The AG and AEG groups performed treadmill running (20 m/min, 60 min/day) five days/week for two weeks. The exercise capacity was significantly higher in the AG and AEG groups compared to other groups. Interestingly, the exercise capacity increased more effectively in the AEG group. The body weight in the EG tended to be slightly lower than that in the other groups. There were no significant changes in the plasma lipids among the groups. However, the gastrocnemius muscle mitochondria content was greater in the EG and AEG groups. These findings show that Echi A administration after short-term endurance training enhances exercise capacity, which was associated with an increase in skeletal muscle mitochondrial content.

## 1. Introduction

In recent years, a large number of novel marine compounds with bioactive properties have been identified with anti-bacterial, anti-protozoal, anti-tuberculosis, anti-viral, anti-diabetic, and anti-inflammatory effects [1]. Marine supplements containing bioactive compounds have been suggested to accelerate recovery following exercise or improve adaptation to training [2]. Sea urchins provide a unique source of marine bioactive compounds that appear to be natural anti-oxidants [3]. In particular, Echinochrome A (Echi A), a natural food-derived pigment isolated from sea urchins has been reported to have anti-oxidant properties and can act as a metal chelator [4]. Recent findings have highlighted a new therapeutic potential of Echi A in the treatment of reduced acetylcholine-related diseases [5]. Furthermore, Echi A and similar hydroxynaphthazarins are reported to exert their bioactive effects at low doses, with moderate or low toxicity in mice *in* *vivo* [6]. Our previous study demonstrated that Echi A can activate the transcription of genes responsible for mitochondrial biogenesis *in vitro* [7] and modulate mitochondrial respiration in the cardiomyoblast H9c2 cell line and isolated rat cardiomyocytes [8]. However, it is unknown whether there are similar mechanisms that influence mitochondrial content or function in skeletal muscle. These possibilities have not been verified by previous studies, although an increase in the mitochondrial content is an important for function of skeletal muscle and increases exercise capacity.

Regular exercise training leads to multiple morphological and cellular adaptations including increased mitochondrial content in skeletal muscle and altered energy metabolism [9]. It is well known that the exercise capacity is a strong predictor of morbidity and mortality [10]; therefore, targeting the exercise capacity is an effective intervention to improve health. In particular, increased mitochondrial content in skeletal muscle as a result of exercise training plays a critical role in the enhancement of exercise capacity [11,12]. There is currently a widespread interest in novel dietary compounds that may support or enhance adaptation to exercise training and improve exercise capacity [13]. Natural marine derived supplements have emerged in recent years as potential ergogenic aids for exercise training. Several studies have reported that Echi A supplementation results in an improvement in mitochondrial function in cardiac muscles [4,5,6,7]. Thus, we hypothesized that Echi A may have ergogenic potential of increasing the exercise capacity in combination with short-term exercise training, which may be partly mediated by an increase in mitochondrial abundance. Moreover, we determined whether Echi A treatment in combination with short-term exercise training altered body weight, muscle mass, and blood lipid levels.

## 2. Results

### 2.1. Effect of Echi A on the Exercise Capacity

**Figure 1:**
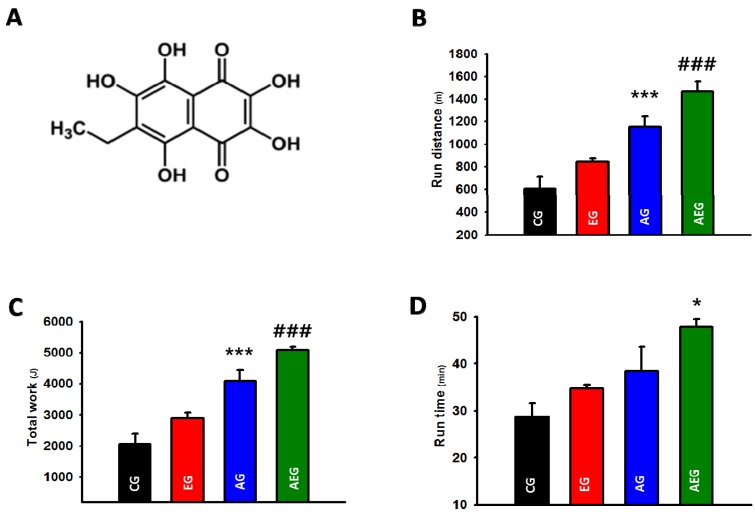
(**A**) Chemical structure of Echi A; (**B**) distance to exhaustion; (**C**) kilojoules work completed during the maximal exercise capacity test; and (**D**) Difference in time to exhaustion between the control group (CG), echinochrome A-treated group (EG), aerobic exercise group (AG), and aerobic exercise group treated with Echi A (AEG). The data are expressed as means ± SE. * *p* < 0.05; CG and EG *vs.* AEG, *** *p* < 0.001; CG and EG *vs.* AG, ^###^ *p* < 0.001; all groups *vs.* AEG.

The chemical structure of Echi A isolated from sea urchin *Saphechinus mirabilis* is shown in Figure 1A. Two weeks of exercise training was sufficient to increase the running distance (*p* < 0.001, Figure 1B) and total work done (*p* < 0.001, Figure 1C) during the maximal exercise capacity test compared to non-exercise trained controls. Importantly, Echi A administration during exercise training resulted in a significantly increased running distance for exhaustion (*p* < 0.001, Figure 1B) and the total work (*p* < 0.001, Figure 1C) completed during the maximal exercise capacity test compared to all other groups. The running time was significantly increased following Echi A administration during exercise training compared to CG and EG (*p* < 0.05, Figure 1D). No changes were observed in the running time, running distance, or total work completed during the maximal exercise capacity test following Echi A administration alone without exercise training compared to the controls (Figure 1B,C).

### 2.2. Effect of Echi A on Body Weight and Skeletal and Cardiac Muscle Weight

**Figure 2:**
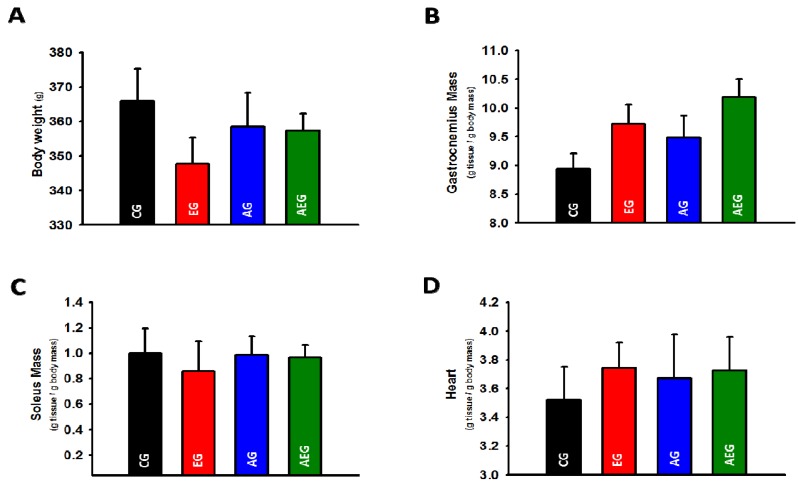
(**A**) Difference in body weight; (**B**) gastrocnemius weight; (**C**) soleus weight and (**D**) heart weight between the control group (CG), echinochrome A-treated group (EG), aerobic exercise group (AG), and aerobic exercise group treated with Echi A (AEG). The data are expressed as means ± SE.

The ratios of the soleus and gastrocnemius muscle weight to the body weight were calculated. Echi A administration during exercise training did not significantly affect body weight, gastrocnemius or soleus muscle weights (Figure 2A–D). The body weight and soleus weight were only slightly lower in rats administered Echi A in comparison with CG (weight approximately 3%). The corresponding difference was lower when compared to the AG and AEG groups. In contrast, the gastrocnemius and heart weights had a slight tendency to increase in groups treated with Echi A.

### 2.3. Effect of Echi A on Plasma Lipids

No significant difference was observed in the plasma lipids with Echi A administration during exercise training. However, exercise training alone also did not significantly alter the plasma lipids (Table 1).

**Table 1:** Biochemical characteristics of the experimental groups.

	CG	AG	EG	AEG
TC (mg/dL)	66.00 ± 9.53 ^1^	56.66 ± 3.51	56.66 ± 6.35	48.66 ± 4.25
TG (mg/dL)	75.66 ± 26.66	43.66 ± 3.84	120.00 ± 51.17	113.33 ± 32.77
LDL-C (mg/dL)	23.66 ± 10.17	11.00 ± 1.15	14.00 ± 6.08	9.33 ± 2.40
HDL-C (mg/dL)	40.33 ± 8.41	36.00 ± 3.21	43.00 ± 4.93	37.33 ± 5.36

^1^ Values are means ± SE. CG: control group, EG: Echi A-treated group, AG: aerobic exercise group, AEG: aerobic exercise group treated with Echi A. TC: total cholesterol, TG: triglycerides, LDL-C: low-density lipoprotein cholesterol, HDL: high-density lipoprotein cholesterol.

### 2.4. Effect of Echi A on Mitochondria in Skeletal Muscle

**Figure 3:**
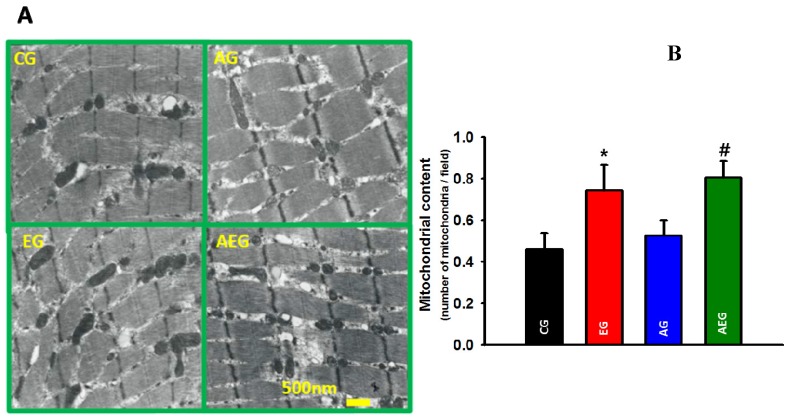
(**A**) Difference in skeletal muscle mitochondria based on transmission electron micrograph imaging between the groups. (**B**) Mitochondrial content was expressed as content of mitochondria/field. Control group (CG), Echi A-treated group (EG), aerobic exercise group (AG), and aerobic exercise group treated with Echi A (AEG). * *p* < 0.05; CG *vs.* EG, ^#^ *p* < 0.05; CG *vs.* AEG. The data are expressed as means ± SE.

We also examined whether Echi A administration during exercise training affected the mitochondria abundance in gastrocnemius muscle using high-resolution transmission electron microscopy at several magnifications*.* We observed a higher mitochondrial abundance in the gastrocnemius muscle of rodents administered Echi A, with (*p* < 0.05) or without exercise training (*p* < 0.05), compared to the untreated non-exercise trained rodents (Figure 3A).

## 3. Discussion

The main findings of the study indicated that Echi A administration during exercise training resulted in a significant improvement in exercise capacity compared to all other groups, which was accompanied by a significant increase in mitochondrial abundance. However, Echi A administration alone without any exercise training did not increase the exercise capacity, but appeared to increase the mitochondrial abundance. Importantly, we demonstrated that Echi A administration appeared to increase mitochondrial abundance in skeletal muscle, but only Echi A administration during exercise training increased the exercise capacity.

Exercise capacity is a tightly controlled process in which the respiration-driven energy supply by mitochondria is balanced by the energy expenditure. It is well recognized that exercise training leads to widespread morphological changes and adaptations in cellular processes that are directly related to mitochondrial abundance and function. In the present study, we show that the mitochondria content in the exercise-trained rodents was greater than that in non-exercise trained controls. Several underlying mechanisms may explain the Echi A-induced improvement in the exercise capacity during short-term training, including mitochondria modulation or antioxidant capacity in skeletal muscle. Previously, *in* *vitro* experiments have shown that Echi A can activate the transcription of genes responsible for mitochondrial biogenesis [7] and modulate mitochondrial respiration in the cardiomyoblast H9c2 cell-line and isolated rat cardiomyocytes [8]. Further studies are necessary to establish whether Echi A, directly or indirectly, modulates mitochondrial function in skeletal muscle *in* *vivo*. Echi A may also potentially modulate antioxidant activity in skeletal muscles. Exercise leads to increased free-radical production, which can cause cellular damage; however, increased antioxidant activity helps remove free radicals *in vitro* in heart cell-lines. This suggests that Echi A possesses antioxidant activity [5], and hence, may reduce the daily accumulation of free radicals and oxidative stress caused by exercise, which in turn may improve recovery. However, further studies on skeletal muscles are necessary to determine whether the Echi A-induced increase in exercise capacity with training may be due to change in the anti-oxidant activity.

It is well known that exercise can reduce body fat and increase the mass of the heart and skeletal muscles. In the present study, exercise tended to inhibit body weight gain in the AG and AEG groups compared to the CG. Interestingly, weight gain was lower in the EG than in other groups, although the difference was not significant (Figure 2A). This is the first study on the ability of Echi A to reduce body weight and increase the mass of heart and gastrocnemius muscle. However, follow-up studies over a longer duration are important to determine whether prolonged treatment with Echi A can inhibit body weight gain with or without exercise training, particularly in animal models of diet-induced obesity.

We acknowledge that there are preliminary findings, and this study has some limitations. Firstly, we used a short-term exercise training duration, which may have limited the effective size between the groups with respect to the main parameters, including exercise capacity and skeletal muscle mitochondria content. Secondly, more mechanistic follow-up experiments are necessary to determine the mechanism underlying the beneficial effect of Echi A administration during exercise training on the enhancement of the exercise capacity, such as changes in mitochondrial oxidative phosphorylation, ATP production, and anti-oxidant activity. Finally, we used healthy rodents in the present study, and, therefore, further studies are required to determine whether Echi A can increase the exercise capacity in rodents with chronic medical conditions, where the exercise capacity is limited, such as in cases who are recovering from a stroke or post-myocardial infarction [14,15].

## 4. Experimental Section

### 4.1. Experimental Design

Sprague-Dawley male rats (age: 8 weeks, *n* = 24) were purchased from the Orient Bio Laboratory Animals (Daejeon, Korea), which is a controlled facility (12:12-h light/dark cycle, 22 °C) and provided with water and food *ad libitum*. The rats were randomly divided into the control group (CG, *n* = 6), Echi A-treated group (EG, *n* = 6), aerobic exercise group (AG, *n* = 6), and aerobic exercise group treated with Echi A (AEG, *n* = 6). All procedures were performed in accordance with Guidelines of Inje Medical University Animal Experimentation Ethics Committee.

### 4.2. Echi A Supplementation and Exercise Training

Echi A was obtained from G.B. Elyakov Pacific Institute of Bioorganic Chemistry, Vladivostok, Russia. The substance was dissolved in phosphate-buffered saline (PBS) 30 min before each exercise training session. The animals in the EG and AEG were injected intraperitoneally with 0.1 mg/kg of Echi A in 300 μL PBS [16]. The animals in the CG and AG groups were administered 1.0 mL PBS intraperitoneally. The exercises were performed on a treadmill (20 m/min, 60 min/day, 5 days/week) for 2 weeks. The treadmill lane has electronic shock bars to ensure that the exercise intensity was consistently maintained. To avoid any potential stress, prior to the experiment, the animals underwent an adaptation period to familiarize the animals with running on the treadmill.

### 4.3. Organ Weight and Biochemical Measurements

After two weeks after exercise training, blood from the heart was drawn into a heparinized tube. The animals were sacrificed by injection of sodium pentobarbital (1 mg/kg) with heparin (300 IU/mL/kg). The body weight was measured daily in the morning before exercise training. The soleus and gastrocnemius muscles were immediately removed and weighed. The ratios of the soleus and gastrocnemius muscle weight to body weight were calculated. Plasma lipids were measured, as previously described [17].

### 4.4. Exercise Capacity Test

To determine the effectiveness of Echi A in improving exercise capacity, all groups underwent an acute incremental exercise capacity test on the treadmill during the last week. The acute incremental exercise capacity test was performed on a treadmill set at a 15° gradient, which started at 10 m/min for 5 min, with increments of 2 m/min every 2 min until exhaustion. The test was terminated when the rats stopped running and were stationary on the electrical shock bar [18]. The total work was calculated as follows: Total work done = force × distance [19].

### 4.5. Transmission Electron Microscopy

The gastrocnemius muscle tissue was cut into small sections (1 mm^3^) and processed in 2.5% glutaraldehyde in 100 mM phosphate buffer at 4 °C for 2 h. The sections were then washed in phosphate buffer and stored at 4 °C or stained as described below. The muscle tissue was post-fixed in 1% osmium tetroxide, dehydrated in an ethanol series, and fixed in epoxy resin (Araldite CY212, Agar Scientific, Elecktron Technology, Stansted, UK). Afterwards, 750 nm sagittal sections were dissected and dyed with toluidine blue for light microscopy analysis. The stained sections were monitored under a Leica DM 6400 microscope (Leica Microsystems, Wetzlar, Germany). For electron microscopy analysis, ultrathin 60–70 nm sections were dissected using an ultramicrotome (LKB Nova, Bromma, Sweden), stained on a 200 mesh copper grid (Agar Scientific, Elecktron Techology, Stansted, UK), and then dyed with uranyl acetate and Reynold’s lead citrate. The grids were visualized with a JEOL 100SX transmission electron microscope (JEOL Ltd., Akishima, Tokyo, Japan), and the images were collected using a photographic film (Kodak 4489, Eastman Kodak Company, Rochester, NY, USA) [20]. The mitochondrial content was determined from the images at 10,000× magnification using Image J software (Version 1.48, NIH, Bethesda, MD, USA) and calculated as mitochondria count/μm^2^. The data displayed mitochondrial contents of 8 micrograph/experimental group/per animal.

### 4.6 Statistical analysis

The data are presented as means ± standard errors. To statistical analysis, we used one-way analysis of the variance (ANOVA) with the Tukey’s *post hoc* test. Statistical significance was observed at a value of *p* < 0.05.

## 5. Conclusions

In conclusion, this is the first study to show that daily intraperitoneal administration of Echi A during short-term endurance training over a period of two weeks improved the exercise capacity compared to control-treated rodents. The beneficial effects of Echi A may be partly due to the enhanced training adaptations, including increased mitochondrial content in skeletal muscle. However, further mechanistic studies are required.
